# Adherence to isoniazid preventive therapy in Indonesian children: A quantitative and qualitative investigation

**DOI:** 10.1186/1756-0500-5-7

**Published:** 2012-01-06

**Authors:** Merrin E Rutherford, Rovina Ruslami, Winni Maharani, Indria Yulita, Sarah Lovell, Reinout Van Crevel, Bachti Alisjahbana, Philip C Hill

**Affiliations:** 1 Centre for International Health, University of Otago, Dunedin, New Zealand; 2 Health Research Unit, Faculty of Medicine, Universitas Padjadjaran, Building 5th Floor, JL Eijkman No. 38 Bandung, Bandung, Indonesia; 3 Bandung community Lung Clinic, Bandung, Indonesia; 4 Department of Preventive and Social Medicine, University of Otago, Dunedin, New Zealand; 5 Department of Medicine, Radboud University Nijmegen Medical Center, Nijmegen, The Netherlands

## Abstract

**Background:**

It is recommended that young child contacts of sputum smear positive tuberculosis cases receive isoniazid preventive therapy (IPT) but reported adherence is low and risk factors for poor adherence in children are largely unknown.

**Methods:**

We prospectively determined rates of IPT adherence in children < 5 yrs in an Indonesian lung clinic. Possible risk factors for poor adherence, defined as ≤3 months prescription collection, were calculated using logistic regression. To further investigate adherence barriers in-depth interviews were conducted with caregivers of children with good and poor adherence.

**Results:**

Eighty-two children eligible for IPT were included, 61 (74.4%) of which had poor adherence. High transport costs (OR 3.3, 95% CI 1.1-10.2) and medication costs (OR 20.0, 95% CI 2.7-414.5) were significantly associated with poor adherence in univariate analysis. Access, medication barriers, disease and health service experience and caregiver TB and IPT knowledge and beliefs were found to be important determinants of adherence in qualitative analysis.

**Conclusion:**

Adherence to IPT in this setting in Indonesia is extremely low and may result from a combination of financial, knowledge, health service and medication related barriers. Successful reduction of childhood TB urgently requires evidence-based interventions that address poor adherence to IPT.

## Background

Increased risk of infection by *Mycobacterium tuberculosis *among children in contact with a sputum smear positive tuberculosis case is well documented [1-4]. Further, risk of progression to disease following infection is particularly high in young children and disease is often severe and disseminated [3,4]. Therefore the WHO recommends routine contact tracing and screening of all child contacts < 5 years followed by six to nine months of isoniazid preventive therapy (IPT) in those where active disease is ruled out [5]. IPT is safe and effective; side effects in children are extremely rare [6-8] and efficacy is over 90% when taken correctly [9]. Nevertheless this recommendation is rarely put in to practice in high burden countries and, where IPT is initiated, adherence is reportedly poor [10,11] and little is known regarding associated adherence barriers [10,12]. Knowledge of such barriers is necessary for targeted interventions.

Indonesia is among the high burden countries [13] that support active contact tracing and IPT as part of their national TB program. In this setting such action has great potential; a recent study from West Java found 51% of actively screened children aged < 9 yrs living with a sputum smear positive case were positive for TB infection.(Rutherford et al. In press) Yet IPT is rarely implemented and current services are centralised to hospitals and specialised clinics. No data are available on adherence levels or associated barriers, with respect to IPT for child case contacts in Indonesia.

Therefore we prospectively assessed adherence rates and risk factors for poor adherence to IPT in a cohort of actively screened Indonesian children < 5 yrs living with a sputum smear positive adult TB case.

## Methods

### Setting and participants

This sub-study was conducted between April 2009 and September 2010 as part of a larger investigation evaluating tools for the diagnosis of latent tuberculosis infection (LTBI).(Rutherford et al. In press) Recruitment was conducted at a community lung clinic in Bandung, Indonesia where all newly diagnosed sputum smear and chest x-ray (CXR) positive adult TB patients (≥15 yrs) with one or more child contacts (household member for at least 3 months; 6 months-9 years old) were invited to bring their children to the clinic to undergo screening. Children who had been diagnosed with TB in the previous year, or whose index case had at least one month of anti-tuberculosis medication, were excluded.

### Screening process

Following informed consent, children were clinically assessed by the study nurse for TB related symptoms. LTBI was evaluated by Quantiferon Gold In-tube assay according to manufactures guidelines, and the tuberculin skin test (TST). Children positive for infection by either test underwent a CXR and a decision was made by a local paediatrician regarding anti-tuberculosis treatment. Children < 5 years who were screened and cleared of disease, were placed on six months IPT in accordance with WHO guidelines and included in this study [5].

### Data collection

Baseline information was gathered from the child's caregiver in their preferred language, including: child demographics and health indictors; disease characteristics of the TB case; household and social factors including socio-economic indicators; and barriers to IPT adherence as hypothesised in the literature. IPT was unsupervised and required caregivers to collect monthly prescriptions which could only be obtained at the study clinic. The first prescription was given directly after clinical assessment. Isoniazid (INH), in powder form, could then be purchased from the clinic or any private pharmacy. Transportation and medication costs were not subsidised. Attendance at each prescription collection was recorded onto a standarised form by study personnel. Levels of adherence were defined as: poor adherence: ≤3 months prescription collection, good adherence: ≥4 months prescription collection. During monthly prescription collection caregivers were questioned about TB related symptoms, medication cost and possible side effects or other difficulties related to medication consumption.

### Qualitative component

Caregivers were purposefully selected to represent children with good adherence (n = 5) and poor-adherence (n = 7). In-depth home-based interviews investigated knowledge of TB aetiology and transmission, the purpose for IPT, TB and IPT beliefs, barriers and facilitators to IPT, and desired program improvements. Interviews were conducted by an experienced qualitative researcher in a language of the participant's choice and lasted between 1-1.5 hrs. They were digitally recorded and transcribed verbatim except where caregivers (n = 3) refused; for these interviews detailed notes were taken. Transcribed interviews were translated into English for analysis.

### Data analysis

Quantitative data were double entered into ACCESS and checked for errors. Adherence status was assessed according to child demographics and TB case characteristics using an extended Wilcoxon rank sum test. Risk factors for poor adherence were investigated using univariate and multivariate logistic regression modelling. Confounding variables were selected for inclusion in multivariate analysis using change in estimate methodology [14]. Data analysis was conducted using STATA version 11. Thematic coding was used to analyse the qualitative interviews.

Ethical clearance was provided by the ethical committee, Faculty of Medicine, Universitas Padjadjaran.

## Results

Of the 260 children recruited for the main study cohort 150 were aged < 5 yrs. Active TB was excluded in 82 children who were then enrolled into this sub-study. The average age was 26.5 months (range 5-59), 49% were male and the majority had evidence of a BCG vaccination (77%). Most children were asymptomatic (68%) and LTBI negative (85%).

### Quantitative analysis

**Figure 1:**
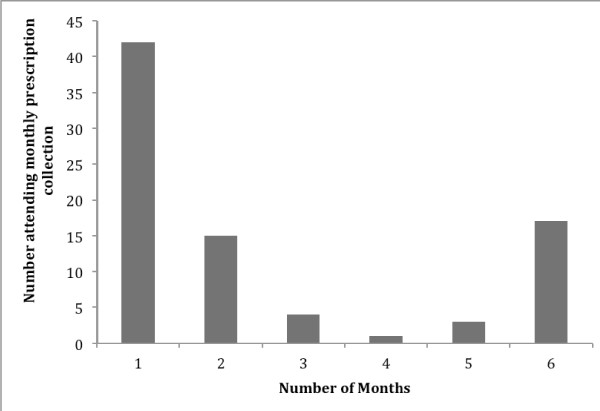
**Distribution of number of months IPT prescription collected**.

Overall 21/82 (25.6% 95% CI 16.6-36.4) and 61/82 (74.4% 95% CI 63.6-83.4) children had good adherence and poor adherence respectively. There was no significant difference between children with good or poor adherence with regards to demographic or TB case factors (Table 1). The average number of months a prescription was collected was 2.5 (95% CI 2.1-2.9). Overall adherence was poor and polarised with caregivers either not returning after collection of the first prescription (51%) or completing the entire course of treatment (17%) (Figure 1).

**Table 1:** Demographic, clinical and TB case details

Variable	Good adherence N = 21 (%)	Poor adherence N = 61 (%)	P-value*
**Sex**			

Male	11 (52)	29 (48)	0.70

Female	10 (48)	32 (52)	

**BCG Scar**			

Yes	17 (81)	46 (75)	0.41

No	3 (14)	7 (11)	

Unknown	1 (5)	8 (16)	

**LTBI at baseline**			

Positive	3 (14)	9 (15)	0.96

Negative	18 (86)	52 (85)	

**Relationship to TB case**			

Child	16 (76)	43 (70)	0.62

Other	5 (24)	18 (30)	

**Sputum smear positivity of contact**			

3 plus	8 (38)	19 (31)	0.79

2 plus	5 (24)	15 (25)	

1 plus	4 (19)	19 (31)	

Scanty	4 (19)	8 (13)	

*Trends calculated by an extension of the Wilcoxon rank-sum test

For barriers related to medication consumption; having medication related problems was seen more frequently in those with poor adherence, while older age was more commonly encountered in those with good adherence, however neither reached significance in univariate analysis (Table 2). For barriers related to medication collection higher transport costs to the clinic (OR 3.3 95% CI 1.1-10.2) and higher medication costs (OR 20.0 95% CI 2.7-414.5) were significantly associated with an increased risk of poor adherence. Children with good adherence tended to be from a household with a higher socio-economic status as estimated by a household income index (total monthly household income divided by number of household members) but this did not reach statistical significance.

**Table 2:** Risk factors for poor adherence

Variable	Good adherence N = 21 (%)	Poor adherence N = 61 (%)	OR (95% CI)	P-value
**Medication consumption barriers**				

**Age (months)**				

0-24	9 (43)	30 (49)	1	

25-60	12 (57)	31 (51)	0.78 (0.29-2.11)	0.62

**Symptoms at baseline**				

No	14 (67)	42 (69)	1	

Yes	7 (33)	19 (31)	0.90 (0.31-2.60)	0.85

**Experienced side effects**				

No	17 (81)	15 (88)	1	

Yes	4 (19)	2 (12)	1.76 (0.28-11.04)	0.54

**Experienced any problem**				

No	18 (86)	15 (79)	1	

Yes	3 (14)	4 (21)	1.60 (0.31-8.30)	0.58

**Medication collection barriers**				

**Income index (Rupiah)**				

0-100,000	1 (6)	8 (16)	1	

> 100,000	17 (94)	42 (84)	0.30 (0.04-2.66)	0.29

**Travel time to clinic (minutes)**				

0-30	11 (52)	33 (54)	1	

> 30	10 (48)	28 (56)	0.93 (0.35-2.52)	0.89

**Transport cost (Rupiah)**				

0-10,000	16 (76)	30 (49)	1	

> 11,000	5 (23)	31 (51)	3.31 (1.08-10.16)	0.04

Medication cost (Rupiah)*				

0-9,000	0 (0)	8 (50)	1	

> 10000	2 (100)	8 (50)	20.00 (2.74-414.45)	0.00

* 0.5 used in place of 0 to calculate OR.

Indonesian Rupiah (IRP) 10,000 = 1USD

### Qualitative analysis

Caregivers interviewed for the qualitative component of this research were predominantly the child's mother (83%) and were aged 26 to 42 years. The children's average age was 2.3 years and 69% were female. Four dominant themes were identified. Quotes illustrating these themes are presented in table 3.

**Table 3:** Example quotes for qualitative themes

Theme	Respondent type	Respondent characteristics	Quote
Access	Good adherence	Mother 30 yrs	Even though I have to buy it (medication) myself, it won't cost me too much because the medicine alone is 20,000 Rp It doesn't cost much.... the fare costs more than the medicine.

Medication-related barriers	Good adherence	Mother 30 yrs	It doesn't happen with my kid. Whenever I tell her to take the medicine she does. But I don't know if I only gave her the powder without mixing it so she doesn't taste the bitterness. I worry she wouldn't take it if she knows it was bitter

	Poor adherence	Mother 32 yrs	He became whiny, must be held by two people. I held him and my sister pushed medicine into his mouth.

Disease and health service experience	Good adherence	Mother 30 yrs	I also felt the benefits of the therapy.... I had constant coughing for 2 months and then it stopped after medication

	Poor adherence	Mother 30 yrs	Because I was afraid she's (nurse) going to be angry with me. I was afraid she would think I didn't prioritize it.

	Poor adherence	Mother 41 yrs	The nurses there didn't tell (the child's) parents that the therapy must be done for 6 months. (The child's) father assumed due to a negative result (TST) the therapy didn't need to be continued

Knowledge and beliefs	Good adherence	Mother 26 yrs	I just wanted (my child) to start therapy immediately because I wanted (my child) to be healthy quickly... So I can stop being worried that something bad would happen to (my child) because there's already preventive medication so I feel secure

	Poor adherence	Mother 42 yrs	I didn't have much time, I had to go here and there... I wasn't concentrating on that (IPT) because I thought (the child) was healthy anyway

### Access

Financial access and distance were frequently mentioned by all respondents as barriers to IPT and resulted directly in poor adherence. Travel cost was the primary access concern and distance as a barrier was frequently mentioned in relation to higher travel costs. For improvements to the existing IPT program, physical access was most often suggested; most respondents suggested IPT should be supplied at the peripheral health clinics to reduce travel cost and distance.

### Medication-related barriers

Medication costs were sighted as marginal compared with other costs involved with IPT, particularly transport. However, where two or more children from the same family were placed on IPT medication costs were more problematic. All respondents mentioned difficulty in medication administration due to the bitter taste of INH. Respondents with poor adherence mentioned personal experiences (Table 3) while respondents with good adherence spoke only in terms of others, expressing a feeling of being fortunate that their children took the medication. Both groups mentioned medication side effects. The absence of side effects acted as a facilitator and only children with poor adherence experienced side effects.

### Disease and health service experience

Respondents' own TB experiences facilitated adherence; respondents with good adherence mentioned a desire for their children to avoid similar disease experiences and expressed confidence in the anti-TB medication they received (Table 3). Such experiences were not described by respondents with poor adherence. Respondents with good adherence frequently mentioned favourable health service experiences, while bad experiences with the health service was noted by respondents with poor adherence and included: fear of staff; lack of or incorrect information provision regarding IPT duration and purpose; and long waiting times.

### Knowledge and beliefs

Knowledge of TB disease aetiology and transmission was low among respondents with poor adherence, while all respondents with good adherence showed correct TB knowledge and an understanding of IPT. Regarding beliefs, respondents with good adherence displayed a desire for health, a preference for prevention over cure and relief at the protection IPT offered. Respondents with poor adherence commonly mentioned that the child was healthy, and such reasoning aided in decisions to stop therapy. Conversely, respondents with good adherence mentioned the need to give medication despite their child's current healthy status.

## Discussion

In this prospective study of IPT adherence and associated barriers in Indonesian children, only 26% of children collected their medication prescription four or more times. Indeed, 51% of all children did not return to the clinic after the first month of medication prescription. Such poor adherence is not unique. Research in TB endemic settings in South Africa found adherence rates for children < 5 years prescribed six months IPT to be between 15-28% [10,11]. In Australia IPT adherence rates among six-year-old children were between 54-74% [15] and in Brazil only 53% of all household contacts completed IPT with 29% being immediately lost to follow-up [16].

While limited by the size of the main study cohort, quantitative analysis did indicate possible risk factors for poor adherence to IPT. These were predominately related to financial barriers for medication collection including cost of medication and transport, and low socio-economic status. Transport cost as a barrier was also frequently mentioned in the qualitative interviews. Financial barriers to IPT adherence have been found elsewhere. In Brazil TB household contacts prescribed IPT, who took two buses to reach the study clinic were 1.8 (95% CI 1.01-3.3) times as likely to be non-adherent compared to those who took only one bus. Authors attributed this to an increase in transport costs [16]. Reduction of cost barriers through provision of IPT services at peripheral health centres was a dominant proposed solution in qualitative interviews.

Quantitative analysis suggested that side effects and other treatment problems might be associated with poor adherence. The importance of side effects was also a theme that emerged from the qualitative interviews. The negative effect of side effects has been sited elsewhere. In Tanzania 14% of HIV positive patients initiated on IPT did not complete therapy due to minor side effects, [17] in Australia 23/32 patients who experienced minor side effects stopped therapy [18] and in Thailand 10/72 HIV positive patients who missed more than one month of IPT did so because of the perceived side effects of INH [19].

We found older age to facilitate IPT adherence. This has been reported elsewhere [12,15]. A South African study found older children were more likely to complete treatment. Authors postulated that difficulty in medication consumption explained this finding. Our study's qualitative findings support this; caregivers frequently expressed that difficulties in medication administration was a significant barrier. Ease of medication administration is especially important where drug regimens are lengthy in duration and for prevention only.

From qualitative interviews IPT knowledge and health beliefs were found to be important. Caregivers of children with good adherence frequently mentioned a desire for health while caregivers of children with poor adherence often expressed a belief that IPT is unnecessary for healthy children. Similarly, in Australia, from interviews with 67 families of children prescribed IPT, only 65% believed their child required therapy [15] while in South Africa reduced risk perception was commonly displayed by parents of non-adherent children [10].

This study has several limitations. The relatively small size limits multivariate analysis and strong inferences are restricted. However, the addition of a qualitative component strengthened conclusions supporting key quantitative findings. Adherence levels could only be measured by whether monthly prescriptions were collected and it cannot be determined whether the medication was actually bought and how much was administered to the child. However it seems reasonable to assume that most caregivers who made the effort to collect their prescription would also purchase and administer the medication, at least after the first month. It is possible that INH was acquired by caregivers at a peripheral health care centre resulting in misclassification and an underestimation of adherence rates. However for this to happen caregivers would have to seek further screening at the peripheral health care centre which seems unlikely. Further, on-going research at peripheral health care centres in this setting shows IPT is very rarely prescribed as a treatment option. Finally, this study was conducted in a single setting, limiting generalizability. Nevertheless the study site is a primary tuberculosis and lung disease referral clinic and the children are likely representative of child case contacts in the city. Further, all eligible child case contacts from consecutively diagnosed sputum smear positive adult TB cases were prospectively and actively screened, reducing selection bias that may exist where only passively screened children initiated on IPT are investigated [10].

## Conclusion

This study highlights poor adherence as a major problem to effective IPT programs in Bandung, Indonesia and indicates financial barriers, poor knowledge, health service failure and difficulty with medication consumption as key barriers. Improving adherence to IPT among child case contacts is crucial to reduce child TB related mortality in high burden countries and to achieve Millennium Goal Four. Efforts to improve adherence levels may include: improving access by disseminated IPT provision; increasing caregiver knowledge of the purpose of IPT through education programmes; and making available affordable child-friendly INH. Such efforts may go a significant way in increasing adherence to IPT in child case contacts in this and similar settings. Research that implements and evaluates interventions to increase adherence levels is now required in high burden low resource settings.

## Competing interests

The authors declare that they have no competing interests.

## Authors' contributions

MR, Study design, data collection, data analysis, primary author. RR, Study design consultant, contribution to final drafts of paper. WH, Data collection, data analysis, contribution to final drafts of paper. IY, Data collection, contribution to final drafts of paper. SL, Qualitative component consultant, contribution to all drafts of paper. RvC, Study design consultant, contribution to all drafts of paper. BA, Contribution to final drafts of paper. PH, Study design, contribution to all drafts of paper. All authors read and approved the final manuscript.
